# Deciduosis of the appendix: a rare cause of acute abdomen during pregnancy (a case report)

**DOI:** 10.11604/pamj.2020.37.316.26728

**Published:** 2020-12-07

**Authors:** Louis Smits, Mieke Van Bockstal, Julie Frezin

**Affiliations:** 1 Department of Abdominal Surgery, Clinique Notre Dame de Grâce, Chaussée de Nivelles, Gosselies, Belgium,; 2 Department of Pathology, Clinique Universitaires Saint-Luc, Avenue Hippocrate, Brussels, Belgium

**Keywords:** Deciduosis, appendicitis, pregnancy, acute abdomen, case report

## Abstract

Appendectomy is the most common non-obstetric surgical intervention in pregnant patients. In rare cases, deciduosis can develop during pregnancy in the appendix and cause inflammation through appendiceal occlusion by ectopic decidua tissue. We report a 28-year-old woman at 32 weeks of pregnancy, who presented at the emergency room with the diagnosis of an acute appendicitis. After successful appendectomy and histopathological examination of the appendectomy specimen, a diagnosis of appendiceal deciduosis with acute appendicitis was established. Here, we discuss the origin of appendiceal deciduosis, as well as its clinical and histopathological presentation.

## Introduction

Decidualization of endometrial tissue is a physiologic phenomenon during pregnancy caused by high progesterone levels. This phenomenon is characterized by hypertrophy of stromal tissue, increased vascular proliferation and glandular secretion [1,2]. Decidual cell groups outside the endometrium are named “ectopic decidua” or “deciduosis” and this condition has first been described by Walker in 1887 [3]. Extrauterine or ectopic decidua is most commonly seen in the ovaries, cervix, uterine serosa and the lamina propria of the fallopian tubes, while peritoneal localization is less frequent. Nevertheless, ectopic deciduosis has been described in the appendix, omentum, liver, diaphragm, paraaortic-pelvic lymph nodes and renal pelvis [1,2,4].

## Patient and observation

A 28-year-old woman, at 32 weeks of pregnancy, without significant medical history, presented to the emergency room with severe abdominal pain in the right flank, evolving during 48 hours. Abdominal palpation revealed tenderness in the right iliac fossa with guarding. Blood test demonstrated an inflammatory syndrome (175 mg/l of C-reactive protein) and neutrophilic hyperleukocytosis.

Two abdominal ultrasounds were performed but were not contributive. Due to lack of clinical improvement and increasing inflammatory parameters, an abdominal CT scan as performed and showed free fluid and inflammation medial to the caecum, next to the gravid uterus. This image was compatible with acute appendicitis. Laparoscopic appendectomy was performed. Conversion to laparotomy was required due to the hemodynamic status of the patient. An inflamed appendix was found just medial to the caecum with necrosis of the tip and a macroscopically healthy base. Free fluid was present in the right iliac fossa. The postoperative period was marked by ileus treated by a nasogastric tube. The patient went into early labor 3 days after surgery and delivered a healthy baby.

**Figure 1:**
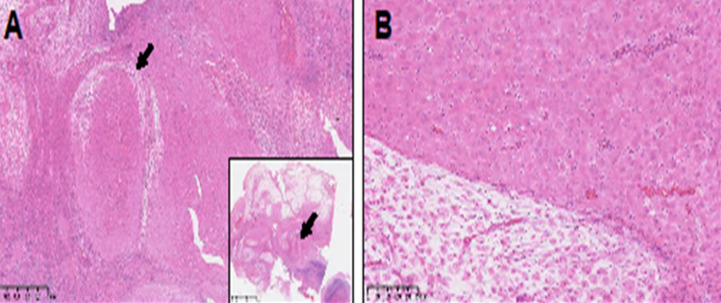
ectopic deciduosis located in the meso of the appendix, constituted by large polygonal nonatypical cells with ample eosinophilic cytoplasm: A) original magnification 50x (inset: 5x); B) original magnification 100x

Macroscopic examination of the appendectomy specimen revealed a 7 cm long appendage with a maximum diameter of 3 cm. The appendiceal wall was significantly thickened. Microscopic examination showed a proliferation of large polygonal decidual cells within the mesoappendix, characterized by abundant eosinophilic cytoplasm and nuclei without any cytonuclear atypia (Figure 1). Mitotic activity was not observed. The extensive appendiceal deciduosis showed strong immunoreactivity for vimentin, without expression of PLAP, broad-spectrum cytokeratins (CK-AE1/AE3), inhibin, CD68, S100 or calretinin. The differential diagnosis of appendiceal deciduosis includes decidualized endometriosis. In the current case, no endometrial glands were identified. Acute inflammation of the appendiceal mucosa was histologically confirmed, suggesting that appendiceal deciduosis might have caused an appendiceal obstruction and subsequent inflammation.

## Discussion

An acute appendicitis occurs in about one under thousand pregnancies and appendectomy is the most common non-obstetric surgical procedure for pregnant patients. But acute appendicitis due to appendicular deciduosis is even more rare [1,2]. The pathogenesis of ectopic decidual reaction is not yet fully understood. Zaystev *et al*. have suggested two theories [5]. The most commonly accepted theory comprises metaplasia of the subcoelomic pluripotent mesenchymal cells under the increasing level of progesterone during pregnancy. The fact that the lesion resolves once the hormonal stimulus ends supports this theory. Another theory is “de novo” development of peritoneal decidual cells [1].

For Buttner *et al*. ectopic decidua is a physiological phenomenon as decidualized tissue was found on 100% of the omentum biopsy in 60 caesarized women, 97% with microscopic involvement and 3% with macroscopic lesions described [2,6]. Finally, another possible explanation for decidualization would be the presence of underlying endometriosis which would decidualize during pregnancy. However, the relationship with endometriosis is uncertain [2]. Usually the condition is asymptomatic and regresses during the weeks after childbirth, but it may reappear in a subsequent pregnancy [1]. Symptomatic ectopic decidua is extremely rare, presenting as appendicitis, intra-abdominal hemorrhage or mechanical ileus [7].

Appendicular deciduosis with acute appendicitis during pregnancy is rare. In the literature, around ten case have been reported [8,9]. It is presumed that, as the endometrium grows during the first trimester of pregnancy in response to gradual increase of progesterone, the ectopic decidua responds in the same fashion and can lead to occlusion of the appendix lumen [10]. Humoral factors may also explain this phenomenon. Because decidua contains high concentrations of prostaglandins witch act as a powerful muscle stimulant, excessive secretion of microsomal acid phosphatase by decidual cells could increase prostaglandin synthesis, causing the appendix muscle wall to contract [8].

On microscopic analysis, decidual cells are generally found under the mesothelium in the subcoelomic mesenchyma or in the fatty tissues. Decidual cells present as large polygonal cells, with homogeneous, eosinophilic cytoplasm and vacuolar degeneration. Mitotic activity and nuclear atypia are absent [1,4]. Immunohistochemistry study allowed to confirm the decidual nature of the tissue (vimentin +, progresteron receptor +) [2], allowing to exclude differential diagnosis (primary or metastatic malignant tumor as deciduoid mesothelioma, abdominal carcinomatosis or metastatic melanoma) [4].

In the present case, the smooth muscle actin and PS100 stains showed no immunoreactivity [2]. The cytokeratine-5, cytokeratine AE1/AE3, calretinin and HBME-1 stains were also negative. This immunohistochemical profile excludes a mesothelioma, a metastatic carcinoma or a metastatic melanoma [1,4]. The definitive treatment of appendicitis caused by deciduosis is surgical and the follow up is generally simple with a regression of the ectopic decidua during the weeks after childbirth.

## Conclusion

We reported a case of acute appendicitis on ectopic decidua during the third trimester of pregnancy. Ectopic decidua is generally asymptomatic and spontaneously regresses within 4-6 weeks after delivery. In rare cases, ectopic decidua can cause acute appendicitis when the appendix is involved. The treatment is surgical and the evolution is favorable in the majority of cases. Definitive diagnosis is performed by histopathological analysis.
